# Variation of B cell subsets with age in healthy Malawians

**DOI:** 10.1371/journal.pone.0254320

**Published:** 2021-07-09

**Authors:** Wilson L. Mandala, Herbert Longwe

**Affiliations:** 1 Academy of Medical Sciences, Malawi University of Science and Technology (MUST), Thyolo, Malawi; 2 The Malawi-Liverpool-Wellcome Trust Clinical Research Programme, Blantyre, Malawi; 3 ICAP at Columbia University in South Africa, Pretoria, South Africa; University of Palermo, ITALY

## Abstract

Although a number of previous studies have shown that different lymphocyte subsets, including B cells, vary with age, how different B cell subsets vary with age in Malawian population has not been shown before. We recruited Malawian participants of different ages and analyzed their venous blood samples for different B cell subsets. We found that both percentage and absolute counts of B cells varied with age peaking in the 7 to 12 months age group. Proportion of naïve B cells was highest in neonates and decreased with age whereas the percentage of memory B cells was lowest in neonates and increased with age. When we zeroed in on the age band within which the proportion of B cells was highest, both classical and activated memory B cells increased with age and the naïve followed the opposite trend. These results provide additional knowledge in our understanding of the dynamics of B cell subsets in individuals of a specific ethnicity as they age.

## Introduction

Several factors are known to affect various immunohaematological parameters in an individual including age, genetics, sex, altitude [1, 2] and social habits such as smoking and dietary patterns [3]. Most of these factors vary depending on the population and geographical area studied [3]. This has the implication that the reference values that have been established and validated for non-adults from one area cannot be used for adult patients in the same area neither can reference values established for a particular ethnic population reliably be used in interpreting haematological parameters of a different ethnic population [2].

For years now flow cytometric analyses have been in use for the diagnosis of various immune deficiencies [4]. The advent of the human immunodeficiency virus (HIV) in the late eighties resulted in the widespread use of flow cytometric analyses not only for diagnosis but also for the monitoring of HIV infection and its progression and also for other infectious diseases, immunologic disorders and malignancies [4–8] and also for establishing reference ranges for leucocyte or lymphocyte subsets based on age, gender or ethnicity in healthy individuals [1–3, 9, 10].

B cells are a part of the cell-mediated immune system which are mostly known for their role in the production of immunoglobulins that are a crucial component of protective immunity to infections [11]. Back in the nineties some researchers discovered that they could distinguish two populations of B cells in the human tonsils and peripheral blood (PB) through expression of CD27 surface antigen [12]. Since then CD27 has become an important marker of human memory B cells. CD27 expression on B cells increases gradually with age and cord blood B cells do not express this marker whereas about 40 percent of adults peripheral blood B cells are CD27^+^ [13]. Subsequently our knowledge of B cell subsets has improved with the advances made in Flow Cytometry. It is now known that B cells in peripheral circulation are made up of about two thirds of naïve (CD19^+^CD21^hi^CD27^-^CD10^-^) which express either switched or unswitched the antibody isotypes, IgG, IgE and IgA, and one-third memory B cells which in turn express switched or unswitched IgM and IgD [11, 14].

More importantly, it is now well established that long lived protective humoral immune response depends on generation of memory B cells that are further subdivided into classical memory (CD19^+^CD27^+^CD21^hi^CD10^-^) and activated memory (CD19^+^CD27^+^CD21^lo^CD10^-^) B cells [15]. Transitional immature B cells area minor population of B cells in peripheral circulations that express an immature phenotype (CD19^+^CD10^+^CD27^-^CD21^lo^) and are less likely to be activated [11]. In addition, a more unique memory B cell subpopulation has now been identified that expresses the surface markers CD19^+^CD27^-^CD21^lo^CD10^-^ and is defined by the expression of the inhibitory receptor Fc-receptor-like-4 (FCRL4) [16]. These atypical memory B cells are functionally distinct from the CD27^+^ memory B cells and reportedly capable of expanding in individuals living in malaria endemic area [15].

Previous reports have shown that leucocyte subsets and lymphocyte subset patterns are mainly affected by ethnicity, gender and environment factors [8, 10]. Thus, the interpretation of the cell-mediated immunity that has been affected by or is responding to an infectious or non-infectious disease is predominantly dependent on having the appropriate normal reference values. We thus conducted this study, firstly to establish normal ranges of absolute counts and percentages (as percentages of total lymphocyte counts) of B cells and their various subsets in healthy Malawians from birth to adulthood. Secondly, having established that the main changes in B cell subsets occur in children aged between 6 and 18 months, we conducted additional set of experiments to determine how different B cell subsets vary with age within this age group.

## Materials and methods

### Participants

For the first part of the study, participants, ranging from newborn babies to adults over 60 years old were recruited from 26^th^ September 2006 up to 15^th^ January 2007 when they came to Ndirande Health Centre for routine health checks, for vaccinations or to give birth. The adults included mothers of the new-born babies whose blood samples were collected on the day of giving birth. Informed written consent was obtained from each adult study participant. In the case of minors, written informed consent was obtained from the parent or guardian of every child. All participants were considered healthy if they had no active disease and had no fever and were not on any medication at the time of recruitment. Blood samples of all participants were screened for HIV and tested for malaria parasitaemia as previously reported [9] and the data of those found to be HIV-infected or parasitaemic were excluded from the final data analysis. Results of this study showed that, unlike other lymphocyte subsets, the proportion of B cell subsets expanded substantially between the ages of 6 and 18 months (S3 Fig in S1 File.). We therefore went further and investigated how the different B cell subsets varied within this age range.

Thus for the second part of the study, a longitudinal approach was adopted. Study participants were confirmed HIV negative children who were known to have been born from HIV negative mothers. Since the second part of the study involved recruitment of minors, informed written consent was obtained from each parent or guardian of every child who met all study eligibility criteria. The children were recruited between August 2012 to February 2014 at aged 6 months then followed up at 12-months stage and then at 18-months stage. At each stage an EDTA blood sample was collected and this was used for HIV tests, malaria parasitaemia determination, Full Blood Count (FBC) and for other immunological assays. During all these visits, the children were seen in the study clinic where they underwent a detailed physical examination.

For both studies, sample size calculation was done based on previous studies’ observation aimed at determining different leucocyte subsets in healthy individuals. These studies had compared sets of fewer than 50 study participants per each age group and identified significant differences in some parameters [1, 2]. Therefore for this study, in order to examine differences between different groups, we planned to recruit 50 participants (25 male and 25 female) for each group in order to have 90% power to detect a difference in mean of 1s, where s is the pooled standard deviation and the 1% significance level is used (to adjust for multiple tests). This calculation is based on use of a Mann-Whitney test and assumes the data are uniformly distributed. Unfortunately, we were not able to achieve these intended sample sizes for both genders and ended up recruiting slightly more of one gender than planned. For the second part of the study the two limiting factors to attaining the intended samples sizes were finding children of the right age at the start of the study (6 months) and the willingness of the mothers to return during the two follow-up stages (12 and 18 months respectively).

### Blood sample analyses

For both the first and the second part of the study, EDTA blood samples were used for full blood counts and for Flow Cytometric analysis. In the case of new born babies in the first part of the study, cord blood was collected from the placenta immediately post-delivery. For the first part of the study, the study participants were grouped into twelve categories based on their ages. Immunophenotyping of blood samples by flow cytometry (Four Colour BD FACSCalibur) was performed as previously reported [9]. Briefly, 25 μL EDTA whole blood sample was labeled with the following monoclonal antibodies (mAbs) in a flow tube: CD27-phycoerythrin (PerCP), and CD19-APC the details of which are provided in S1 Table in S1 File. A separate tube was included which had a similar volume of whole blood sample but was labeled with the following isotype control mAbs: Anti-moGI-FITC, Anti-moGI-PE, Anti-moGI-PerCP and Anti-moGI-APC. The details of these isotype control antibodies are also provided in S1 Table in S1 File.

Red cells were then lysed by using FACSlysing solution (Becton Dickinson) and samples washed with PBS. Sample cells were acquired by using a Four-Colour FACSCalibur flow cytometer (Becton Dickinson). Routine calibration and internal quality assurance of the instrument was performed by using Calibrite Beads and FACSComp software (both Becton Dickinson). Sample data were analyzed by using CellQuest software (Becton Dickinson). Total lymphocytes were identified by light scatter characteristics and the following lymphocyte subpopulations identified as percentages of the lymphocyte gate: B cells (CD19+), memory B cells (CD19+CD27+) and naïve B cells (CD19+CD27-) as depicted in the Gating strategy in S1 Fig in S1 File. Proportion of B cells, memory B cells and naïve B cells were determined from subpopulation percentages whereas the absolute counts of the same cell subsets were calculated from the flow readout percentages and total lymphocyte counts from the hematologic analyzer.

For the second part of the study, 25 μL of the EDTA whole blood sample was incubated with anti CD19 APC, anti CD21 PE-cy5 (all from BD Pharmingen, San Jose, California), anti CD10 FITC and anti CD27 PE (eBiosciences, San Diego, California) details of which are provided in S1 Table in S1 File. Labeled blood samples were then lysed, washed as explained for the first group and analysed by flow cytometry (Nine colour Cyan (Beckman Coulter) and the gating strategy shown in S2 Fig in S1 File was used for classifying different B cell subsets. B cells were identified as CD19^+^ and subpopulations of naïve as CD19^+^CD21^hi^CD27^-^CD10^-^, classical memory as CD19^+^CD27^+^CD21^hi^CD10^-^, activated memory as CD19^+^CD27^+^CD21^lo^CD10^-^, atypical memory CD19^+^CD27^-^CD21^lo^CD10^-^ and immature transitional as CD19^+^CD10^+^CD27^-^CD21^lo^.

### Statistical analysis

For the first part of the study, the statistical analysis involved dividing the study participants into 12 age groups as indicated in Table 3. Kruskal-Wallis Test was used to assess if there were statistically significant differences in the medians of B cell subsets’ counts or proportions amongst the various age groups. Considering that comparisons for more than two groups were made, between-age-group comparisons of the different B cell subsets were assessed using Dunn’s multiple comparison test and where a *p* value of <0.0125 was obtained, the difference between specific age groups was considered statistically significant.

**Fig 1:**
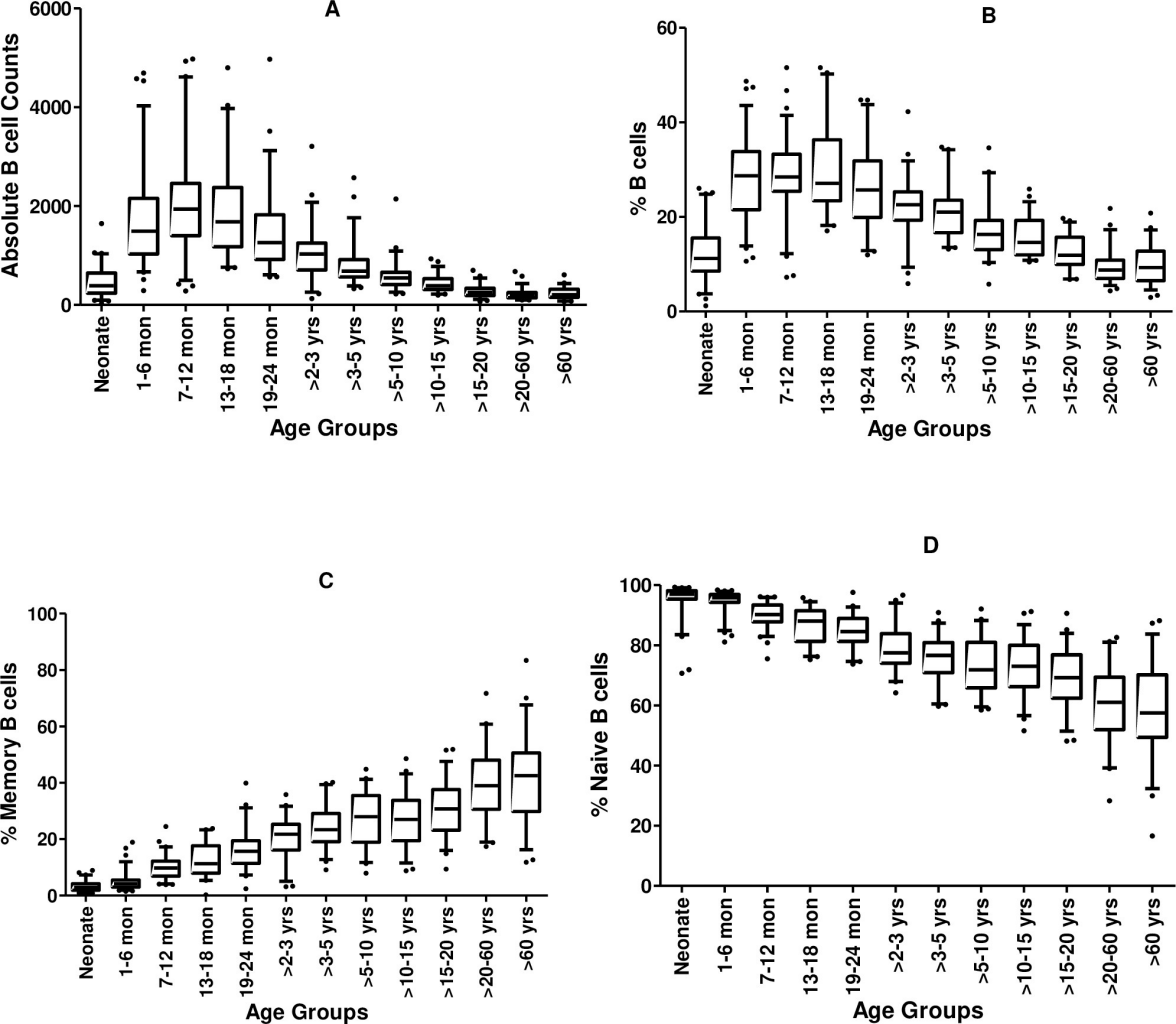
Medians and 10^th^ and 90^th^ percentiles of various subsets of B cells in different age groups. (A) Total B cells expressed as absolute cell counts, (B) Total B cells expressed as percentage of total lymphocytes, (C) Percentage of memory B cells (calculated from total B cells) and (D) Percentage of naïve B cells (calculated from total B cells).

Analysis of variance was conducted using Statistical Package for Social Sciences (SPSS), version 14 (Norusis, SPSS, Chicago, IL, USA). The results in Table 3 are presented as medians and 10^th^ and 90^th^ percentiles of the absolute counts and percentage of B cell subsets population analysed after age stratification. Since we observed that there were no significant differences between male and female participants for any of the various B cell subsets (absolute counts or proportions) (S2 Table in S1 File) therefore data for male and female participants in each age group were combined and analysed together. For the box whisker plots in Fig 1, the top, bottom and line through the middle of the box correspond to the 75^th^, 25^th^ and 50^th^ percentiles (median), respectively. The whiskers extend from 10^th^ percentile at the bottom to 90^th^ percentile at the top. Outliers are presented as filled circles.

For the second part of the study, GraphPad Prism 6 (GraphPad, California, USA) and STATA (StataCorp, Texas, USA) were used for carrying out the statistical analysis and constructing the graphical presentation of the results. Kruskal-Wallis Test was used to assess if there were statistically significant differences in the medians of B cell subsets’ counts or proportions between the three time points. Considering that comparisons for three time points were made, comparisons of the different B cell subsets between the set time points were assessed using Dunn’s multiple comparison test and where a *p* value of <0.0125 was obtained, the difference between specific time points was considered statistically significant.

### Ethics approval

Both the first and the second part of the study were reviewed and approved by the College of Medicine Research Ethics Committee (COMREC) and were assigned the following Protocol Numbers P.01/02/176 and P.05/10/954 respectively. Individual written informed consent was obtained from either the participating adults or from the parents or guardians of all the children who participated in the study.

## Results

For the first part of the study, a total of 715 participants were recruited, and these ranged from neonates to adults over 60 years old (Table 1). Slightly more female participants [399 (56%)] took part in the study than male participants [316 (44%)]. The 55 participants in the group aged 60 years or more had a media age of 66 years with the age ranging from 60 to 92 years.

**Table 1:** Age distribution of HIV-negative study participants according to gender.

Group Number	Age Range	Number of Female Participants	Number of Male Participants	Totals (Female and Male)
1	Neonates	37	24	61
2	1 to 6 months	44	24	68
3	7 to 12 months	31	35	66
4	13 to 18 months	15	27	42
5	19 to 24 months	16	33	49
6	>2 to 3 years	20	28	48
7	>3 to 5 years	22	29	51
8	>5 to 10 years	30	26	56
9	>10 to 15 years	36	17	53
10	>15 to 20 years	29	29	58
11	>20 to 60 years	33	20	53
12	>60 years	31	24	55
		**399**	**316**	**715**

There were no significant differences between male and female participants for the frequency and absolute counts of various B cell subsets (S2 Table in S1 File). As such we combined data for female and male participants for each age range for subsequent statistical analyses for the first part of the study. Medians, 10^th^ and 90^th^ percentiles of the data for B cell counts and percentages are presented in Table 2 and in Fig 1.

**Table 2:** Medians and 10^th^ and 90^th^ percentiles of absolute B cell counts per μl of blood and for proportion of B cells, memory and naïve B cells in healthy participants of different ages.

Age	B Cell Counts/μL	B cells (%)	Memory B cells (%)	Naïve B cells (%)
Neonates (n = 61)	387 (147–899)	11.24 (4.86–19.40)	2.84 (1.16–5.56)	97.03 (91.27–98.84)
1–6 months (n = 68)	1,494 (739–2,882)	28.71 (16.28–38.10)	4.16 (2.52–8.59)	95.83 (91.25–97.41)
7–12 months (n = 66)	1,937 (988–3,130)	28.46 (17.60–36.92)	9.75 (4.97–16.45)	90.25 (83.90–95.03)
13–18 months (n = 42)	1,682 (931–3,431)	27.11 (20.63–46.10)	11.30 (5.92–20.64)	88.06 (77.84–93.12)
19–24 mon (n = 49)	1,261 (701–2,382)	25.70 (15.80–39.24)	15.67 (9.44–25.40)	84.56 (75.66–90.56)
>2–3 years (n = 48)	1,030 (457–1,558)	22.57 (13.23–27.43)	21.72 (10.83–28.37)	77.51 (69.60–89.17)
>3–5 years (n = 51)	686 (433–1,393)	21.00 (15.43–31.46)	23.39 (14.22–34.66)	76.61 (65.34–85.78)
>5–10 years (n = 56)	545 (304–857)	16.30 (11.32–25.50)	27.98 (14.26–40.40)	71.84 (61.38–85.62)
>10–15 years (n = 53)	388 (241–672)	14.60 (10.90–21.64)	27.00 (15.68–39.60)	73.00 (60.40–84.18)
>15–20 years (n = 58)	253 (144–454)	11.88 (7.51–18.23)	30.72 (17.55–45.36)	69.28 (54.11–82.45)
>20–60 years (n = 53)	194 (109–293)	8.79 (5.86–13.89)	38.97 (25.52–56.25)	61.03 (43.75–74.48)
>60 years (n = 55)	208 (122–421)	9.28 (5.35–15.53)	42.50 (21.69–63.22)	57.50 (36.78–78.31)

The B lymphocyte subset showed a similar trend for both percentages and absolute counts.

Absolute B cell counts (Table 2 and Fig 1A) were low (387 cells/μl) in neonates, increased to the highest value (1,937 cells/μl) in the 7-to-12-moths age group, followed by a decrease reaching the lowest value (194 cells/μl) in the 20 to 60 years age group. A similar trend was observed when the B cells were presented as a percentage of the total lymphocytes (Table 2 and Fig 1B). The proportion of B cells was low in neonates (11.24%), increased gradually with age reaching the highest percentage in 7-to-12 months age group (28.46%) followed by a gradual decrease with the lowest percentage (9.28%) observed in the age group of 60 years and above.

The percentage of memory B cells were low in neonates (2.84%) and increased gradually with age with the maximum percentage (42.50%) observed in those aged 60 years and above (Table 2 and Fig 1C). An opposite trend was observed for the percentage of naïve B cells (Table 2 and Fig 1D). These were highest in neonates (97.03%) and decreased gradually with age with the lowest median percentage (57.50%) observed in those aged 60 years and above.

Having noticed that the main changes in the proportion and absolute counts of the B cells occur between 6 and 18 months (Fig 1A and 1B and S3 Fig in S1 File), we then focussed on this age band and adopted a longitudinal approach in order to identify the specific changes that occur to a few more subsets of B cells.

**Fig 2:**
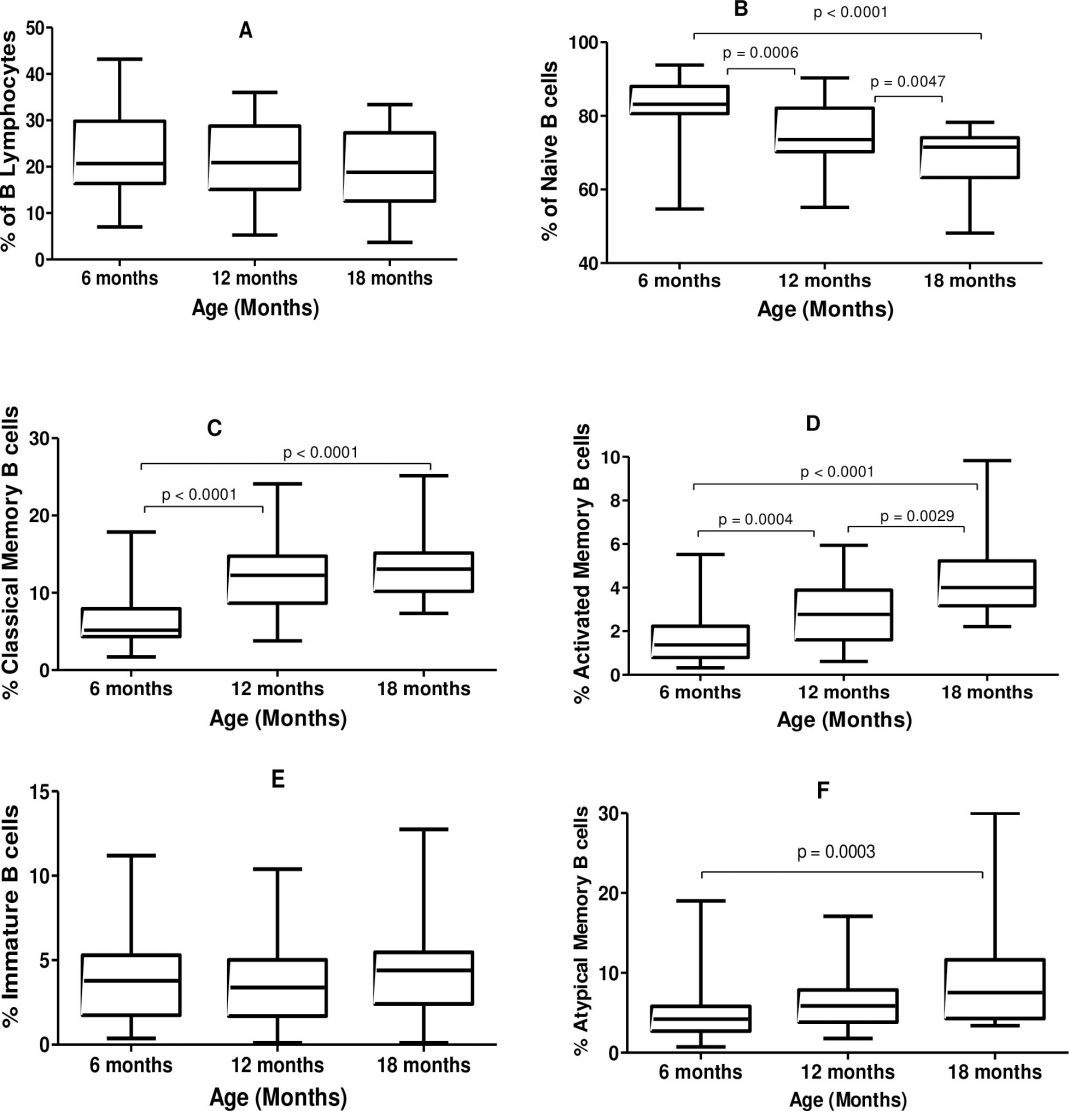
Median proportions of naïve and memory CD19^+^ B cell subsets in healthy children at different ages. (A) Percentage of B lymphocytes, (B) proportions of naïve, (C) classical memory, (D) activated memory, (E) immature transitional and (F) atypical memory B cells.

Just as was the case with the first cohort of the study, the percentage of B cells (as a percentage of total lymphocytes) did not significantly differ between 6, 12 and 18 months points (Table 3, Fig 2A). However the percentage of naïve B cells (as a percentage of total B cells) was significantly higher at 6 months stage (83.2%) compared to that at 12 months (73.6%) and 18 months (71.5%) (Table 3, Fig 2B).

**Table 3:** Median proportion of B cell subsets in the study participants at different age points (6, 12 and 18 months).

Time Points	Proportion of CD19^+^ B cells subsets (%)					
	Total CD19^+^	Naïve	Classical memory	Activated memory	Atypical memory	Immature transitional
**6-months (n = 29)**	20.7 (16.8–29.1)	83.2 (80.7–87.9)	5.26 (4.59–7.85)	1.37 (0.85–2.16	4.21 (2.75–5.65)	3.78 (2.21–5.09)
**12-months (n = 29)**	20.9 (15.2–27.4)	73.6 (70.6–81.9)	12.3 (8.68–14.4)	2.77 (1.64–3.78)	5.86 (3.84–7.77)	3.38 (1.70–4.54)
**18-months (n = 29)**	18.8 (12.6–27.3)	71.5 (63.2–74.0)	13.0 (10.2–15.2)	4.0 (3.17–5.22)	7.53 (4.28–11.6)	4.39 (2.41–5.47)

As expected, the percentage of classical memory B cells was significantly lower at 6 months (5.26%) compared to 12 months (12.3%) and at 18 months (13.0%) (Table 3, Fig 2C) and a similar trend was observed for activated memory B cells (Fig 2D). There were no differences in the proportion of immature transitional B cells between any of the three age stages (Fig 2E) whereas the percentage of atypical memory B cells was significantly higher at 18-months age point than at 6-months point (Fig 2F).

## Discussion

B cells play a fundamental role in human immune response against infections since, once they differentiate to plasma B cells, they serve as antibody producing cells. Thus establishing how these cells are affected by age in healthy individuals is important as these values can then be used as a reference point in explaining any variation that might occur due to infection or vaccination [17–19].

Over the years various investigators have conducted studies with the aim of determining the normal B cell subset ranges for their population. Table 4 summarises the distribution of B cell subsets in study participants from various countries.

**Table 4:** Medians (10^th^ and 90^th^ percentiles) of proportions and absolute counts of B cells in study participants from different countries in the age range of 18 to 40 years.

		% CD19+ B cells	CD19+ B cells/μl
1	Our study (n = 53)	11.88 (7.51–18.23)	253 (144–454)
2	Ethiopia [1] (n = 51)	11.0 (4.0–24.0)	181 (56–436)
3	Tanzania [17] (n = 214)	13.0 (6.0–21.0)	253 (88–654)
4	Holland [20] (n = 678)	NA	290 (110–670)
5	Germany [21] (n = 100)	NA	220 (80–490)
6	Germany [22] (n = 32)	9.2 (7.2–11.2)	199 (169–271)
7	USA [10] (n = 784)	NA	300 (110–570)
8	Cuba [23] (n = 129)	25.6 (5.4–49.5)	452 (114–1,491)
9	China [24] (n = 135)	13.00 (8.84–17.76)	316 (203–476)
10	Singapore [25] (n = 38)	12.0 (10.0–15.0)	200 (100–200)
11	Korea [26] (n = 294)	10.43 (10.0–10.86)	203 (191–214)
12	Saudi Arabia [27] (n = 30)	13.9 (5.0–27.0)	298 (110–730)

The results of our study compared well with the results of some similar studies conducted in other countries with almost identical values for B cell counts for participants in Tanzania and Germany (Table 4). Although we [28] and others [19] have previously shown that ethnicity does have an effect on some lymphocyte subsets, the results presented in Table 4 seem to suggest that, with the exception of the Cuban study [23], proportion and cell counts of B cell subsets do not differ substantially between the different ethnic groups. The higher proportion and absolute counts for B cells observed in the Cuban population was explained as a possible result of continuous exposure to viral and other infections [23].

CD27 is a marker of primed memory cells and its engagement promotes the differentiation of memory B cells into plasma cells [29]. Evaluation of maturation-specific phenotypes in peripheral blood is useful in providing evidence for prior antigen exposure and assessing the resulting lymphocytes homing patterns [30]. Previously some investigators [31] demonstrated an increase of CD27+ B cells and a decrease of CD27- B lymphocytes in elderly. These age-related changes were also observed in our study.

Recent studies on how B cell subsets vary with age [19, 32–34] have also shown similar trends to those observed in our study in terms of naïve B cells (decreasing with age) classical memory B cells and activated memory B cells (increasing with age). The observation made in our study showing significantly higher percentage of atypical memory B cells at 18 months compared to 6 months is fascinating and needs to be studied further.

Similar to other studies [21, 26, 35], we had previously shown that there were no significant differences between male and female participants for the frequency and absolute counts of various lymphocyte subsets including B cells [9] although the other studies had found some significant differences in CD4+ and CD8+ T cells [26, 35]. Further analysis of the data for the first part of this study confirmed this observation for B cells (S2 Table in S1 File).

Although social habits such as smoking and dietary patterns can potentially affect the proportion of different lymphocyte subsets [3, 19], the inclusion and exclusion criteria for recruitment of study participants for this work were stringent enough to ensure that all study participants were non-smokers and were not in any way malnourished or overweight or presenting with any known disease at the time of recruitment.

The main limitation of the first part of the study was that, due to the flow cytometer we had access to at the time (Four-colour BD FACS Calibur), we could only study the two main subsets of B cells (naïve and memory) but could not include the other subsets which we were able to study in the second cohort with the use of the Nine-Colour Cyan flow cytometer. The second cohort’s main limitation was the reduced sample sizes and the limited follow up period of three time points (6, 12 and 18 months). The next obvious study to build on these results should aim at following up the children from 1 month to 60 months with several time points in between and analyse the blood samples for even more B cell subsets such as switched and non-switched memory B cells, CD27-negative memory B cells, transitional B cells as well as CD21lowCD38low B cells as some [22] have done.

## Conclusion

We have shown that B cell subsets in Malawians vary with age but not by gender. The Proportion and absolute counts of B cells reach a peak in the 7-to-12-months age group and were lowest in those aged 60 years and above. These observations could be useful in the interpretation of the natural variation in B cell subsets in otherwise healthy individuals and provide insight on how they respond to infection and vaccination in this setting.

## Supporting information

S1 File Supplementary S1-S3 Figs and Supplementary S1 and S2 Tables. (DOCX)

## References

[1] Tsegaye A., Wolday D., Otto S., Petros B., Assefa T., Alebachew T., et al. Immunophenotyping of blood lymphocytes at birth, during childhood, and during adulthood in HIV-1-uninfected Ethiopians. Clin Immunol 2003; 109:338–346. doi: 10.1016/j.clim.2003.08.008 14697749

[2] Menard D., Mandeng M. J., Tothy M. B., Kelembho E. K., Gresenguet G., and Talarmin A. Immunohematological reference ranges for adults from the Central African Republic. Clin Diagn Lab Immunol 2003; 10:443–445. doi: 10.1128/cdli.10.3.443-445.2003 12738646 PMC154963

[3] Uppal S. S., Verma S., and Dhot P. S. Normal values of CD4 and CD8 lymphocyte subsets in healthy Indian adults and the effects of sex, age, ethnicity, and smoking. Cytometry B Clin Cytom 2003; 52:32–36. doi: 10.1002/cyto.b.10011 12599179

[4] Oliveira JB, Notarangelo LD, Fleisher TA. Applications of flow cytometry for the study of primary immune deficiencies. Curr Opin Allergy Clin Immunol 2008; 8:499–509. doi: 10.1097/ACI.0b013e328312c790 18978463

[5] Janossy G, Shapiro H. Simplified cytometry for routine monitoring of infectious diseases. Cytometry B Clin Cytom 2008; 74 Suppl 1:S6–10. doi: 10.1002/cyto.b.20405 18228555

[6] Ek T, Josefson M, Abrahamsson J. Multivariate analysis of the relation between immune dysfunction and treatment intensity in children with acute lymphoblastic leukemia. Pediatr Blood Cancer 2011; 56:1078–87. doi: 10.1002/pbc.23043 21344616

[7] van Tilburg CM, van Gent R, Bierings MB, Otto SA, Sanders EA, Nibbelke EE, et al. Immune reconstitution in children following chemotherapy for haematological malignancies: a long-term follow-up. Br J Haematol 2011; 152:201–10. doi: 10.1111/j.1365-2141.2010.08478.x 21114483

[8] Ananworanich J, Apornpong T, Kosalaraksa P, Jaimulwong T, Hansudewechakul R, Pancharoen C, et al. Characteristics of lymphocyte subsets in HIV-infected, long-term nonprogressor, and healthy Asian children through 12 years of age. J Allergy Clin Immunol 2010; 126:1294–301 e10. doi: 10.1016/j.jaci.2010.09.038 21134574 PMC3004741

[9] Mandala WL, MacLennan JM, Gondwe EN, Ward SA, Molyneux ME, MacLennan CA. Lymphocyte subsets in healthy Malawians: implications for immunologic assessment of HIV infection in Africa. J Allergy Clin Immunol 2010; 125:203–8. doi: 10.1016/j.jaci.2009.10.010 19944455 PMC2887487

[10] Shearer WT, Rosenblatt HM, Gelman RS, Oyomopito R, Plaeger S, Stiehm ER, et al. Lymphocyte subsets in healthy children from birth through 18 years of age: the Pediatric AIDS Clinical Trials Group P1009 study. J Allergy Clin Immunol 2003; 112:973–80 doi: 10.1016/j.jaci.2003.07.003 14610491

[11] Caraux A, Klein B, Paiva B, Bret C, Schmitz A, Fuhler GM, et al. Myeloma Stem Cell Network. Circulating human B and plasma cells. Age-associated changes in counts and detailed characterization of circulating normal CD138- and CD138+ plasma cells. Haematologica. 2010 Jun;95(6):1016–20. doi: 10.3324/haematol.2009.018689 20081059 PMC2878802

[12] Maurer D., Fischer G. F., Fae I., Majdic O., Stuhlmeier K., Von Jeney N., et al. IgM and IgG but not cytokine secretion is restricted to the CD27+ B lymphocyte subset. J Immunol 1992; 148:3700–3705. 1318333

[13] Agematsu K., Hokibara S., Nagumo H., and Komiyama A. CD27: a memory B-cell marker. Immunol Today 2000; 21:204–206. doi: 10.1016/s0167-5699(00)01605-4 10782048

[14] Moir S, Fauci AS. B cells in HIV infection and disease. Nat Rev Immunol. 2009; 9(4):235–45. doi: 10.1038/nri2524 ; PMCID: PMC2779527. 19319142 PMC2779527

[15] Weiss GE, Crompton PD, Li S, Walsh LA, Moir S, Traore B, et al. Atypical memory B cells are greatly expanded in individuals living in a malaria-endemic area. J Immunol. 2009; 183(3):2176–82. doi: 10.4049/jimmunol.0901297 Epub 2009 Jul 10. 19592645 PMC2713793

[16] Moir S., Ho J., Malaspina A., Wang W., DiPoto A. C., O’Shea M. A., et al. Evidence for HIV-associated B cell exhaustion in a dysfunctional memory B cell compartment in HIV-infected viremic individuals. The Journal of experimental medicine, 2008; 205(8), 1797–1805. doi: 10.1084/jem.20072683 18625747 PMC2525604

[17] Urassa W. K., Mbena E. M., Swai A. B., Gaines H., Mhalu F. S., and Biberfeld G. Lymphocyte subset enumeration in HIV seronegative and HIV-1 seropositive adults in Dar es Salaam, Tanzania: determination of reference values in males and females and comparison of two flow cytometric methods. J Immunol Methods 2003; 277:65–74. doi: 10.1016/s0022-1759(03)00174-1 12799040

[18] Embree J., Bwayo J., Nagelkerke N., Njenga S., Nyange P., Ndinya-Achola J., et al. Lymphocyte subsets in human immunodeficiency virus type 1-infected and uninfected children in Nairobi. Pediatr Infect Dis J 2001; 20:397–403. doi: 10.1097/00006454-200104000-00006 11332664

[19] Noren Hooten N., Longo D.L., Evans M.K. Age- and Race-Related Changes in Subpopulations of Peripheral Blood Lymphocytes in Humans. In: Fulop T., Franceschi C., Hirokawa K., Pawelec G. (eds) 2019. Handbook of Immunosenescence. Springer, Cham. 10.1007/978-3-319-99375-1_85

[20] Kassu A., A. Tsegaye B., Petros D., Wolday E., Hailu T., Tilahun B., et al. Distribution of lymphocyte subsets in healthy human immunodeficiency virus-negative adult Ethiopians from two geographic locales. Clin Diagn Lab Immunol. 2001: 8:1171–1176. doi: 10.1128/CDLI.8.6.1171-1176.2001 11687459 PMC96245

[21] Jentsch-Ullrich K., Koenigsmann M., Mohren M., and Franke A. Lymphocyte subsets’ reference ranges in an age- and gender-balanced population of 100 healthy adults: a monocentric German study. Clin Immunol. 2005: 116:192–197. doi: 10.1016/j.clim.2005.03.020 15993366

[22] Morbach H, Eichhorn EM, Liese JG, Girschick HJ. Reference values for B cell subpopulations from infancy to adulthood. Clin Exp Immunol. 2010;162(2):271–9. doi: 10.1111/j.1365-2249.2010.04206.x 20854328 PMC2996594

[23] Kokuina E, Breff-Fonseca MC, Villegas-Valverde CA, Mora-Díaz I. Normal Values of T, B and NK Lymphocyte Subpopulations in Peripheral Blood of Healthy Cuban Adults. MEDICC Rev. 2019; 21(2–3):16–21. . 31373580 10.37757/MR2019.V21.N2-3.5

[24] Ding Y, Zhou L, Xia Y, Wang W, Wang Y, Li L, et al. Reference values for peripheral blood lymphocyte subsets of healthy children in China. J Allergy Clin Immunol. 2018;142(3):970–973.e8. doi: 10.1016/j.jaci.2018.04.022 29746882

[25] Lee B. W., Yap H. K, Chew F. T., Quah T. C., Prabhakaran K., Chan G. S. et al. Age- and sex-related changes in lymphocyte subpopulations of healthy Asian subjects: from birth to adulthood. Cytometry. 1996: 26:8–15. doi: 10.1002/(SICI)1097-0320(19960315)26:1&lt;8::AID-CYTO2&gt;3.0.CO;2-E 8809475

[26] Choi J, Lee SJ, Lee YA, Maeng HG, Lee JK, Kang YW. Reference values for peripheral blood lymphocyte subsets in a healthy Korean population. Immune Netw. 2014;14(6):289–95. doi: 10.4110/in.2014.14.6.289 25550695 PMC4275386

[27] Shahabuddin S., Al-Ayed I., Gad El-Rab M. O., and Qureshi M. I. Age-related changes in blood lymphocyte subsets of Saudi Arabian healthy children. Clin Diagn Lab Immunol. 1998: 5:632–635. doi: 10.1128/CDLI.5.5.632-635.1998 9729529 PMC95633

[28] Mandala WL, Ananworanich J, Apornpong T, Kerr SJ, MacLennan JM, Hanson C. et al. Control lymphocyte subsets: can one country’s values serve for another’s? J Allergy Clin Immunol. 2014; 134(3):759–761.e8. doi: 10.1016/j.jaci.2014.06.030 25171870 PMC4150016

[29] Colonna-Romano G, Bulati M, Aquino A, Scialabba G, Candore G, Lio D, et al. B cells in the aged: CD27, CD5, and CD40 expression. Mech Ageing Dev 2003; 124(4):389–393 doi: 10.1016/s0047-6374(03)00013-7 12714244

[30] Bisset L. R., Lung T. L., Kaelin M., Ludwig E., and Dubs R. W. Reference values for peripheral blood lymphocyte phenotypes applicable to the healthy adult population in Switzerland. Eur J Haematol 2004; 72:203–212. doi: 10.1046/j.0902-4441.2003.00199.x 14962239

[31] Colonna-Romano G, Aquino A, Bulati M, Di Lorenzo G, Listì F, Vitello S, et al. Memory B cell subpopulations in the aged. Rejuvenation Res. 2006; 9(1):149–52. doi: 10.1089/rej.2006.9.149 . 16608412

[32] Berrón-Ruíz L., G. López-Herrera C.E. Ávalos-Martínez C., Valenzuela-Ponce E. Ramírez-SanJuan G. Santoyo-Sánchez F., et al. Variations of B cell subpopulations in peripheral blood of healthy Mexican population according to age: Relevance for diagnosis of primary immunodeficiencies. Allergol Immunopathol (Madr). 2016; 44:571–9 doi: 10.1016/j.aller.2016.05.003 27780620

[33] Yi J. S., Rosa-Bray M., Staats J., Zakroysky P., Chan C., Russo M. A., et al. Establishment of normative ranges of the healthy human immune system with comprehensive polychromatic flow cytometry profiling. PLoS One, 2019; 14(12), e0225512. doi: 10.1371/journal.pone.0225512 31825961 PMC6905525

[34] Frasca D, Blomberg BB. Aging affects human B cell responses. J Clin Immunol. 2011;31(3):430–5. doi: 10.1007/s10875-010-9501-7 21318330 PMC5560853

[35] Yan J, Greer JM, Hull R, et al. The effect of ageing on human lymphocyte subsets: comparison of males and females. Immun Ageing. 2010;7:4. doi: 10.1186/1742-4933-7-4 20233447 PMC2858100

