# Supplementary material for: Variation of B cell subsets with age in healthy Malawians
Source: PLoS One. 2021 Jul 9;16(7):e0254320. doi: 10.1371/journal.pone.0254320 (PMC8270464; doi:10.1371/journal.pone.0254320)
Supplement: S1 File — (DOCX) [file pone.0254320.s001.docx]

**Variation of B cell Subsets with age in Healthy Malawians**

Wilson L. Mandala^1,2^ and Herbert Longwe^2,3^

^1^Academy of Medical Sciences, Malawi University of Science and Technology (MUST), Thyolo, Malawi

^2^The Malawi-Liverpool-Wellcome Trust Clinical Research Programme, Blantyre, Malawi

^3^ICAP at Columbia University in South Africa, Pretoria, South Africa

**SUPPLEMENTARY FIGS**

**S1 Fig**: **Gating strategy for the first part of the study**. Flow cytometer dot plots for (A) forward scatter versus side scatter plot showing R1 gate for lymphocytes and (B) CD27+ (memory) and CD27- B (naïve) cells. CD19+ cells were defined as B lymphocytes, CD19+CD27- as naïve B cells and CD19+CD27+ as memory B cells.


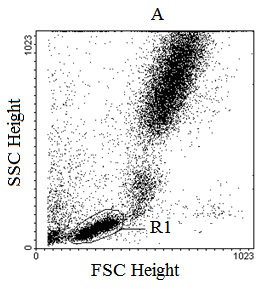

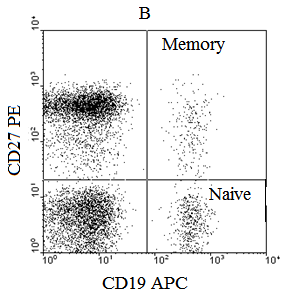


**S2 Fig: Flow cytometry gating strategy for describing B cell subsets using different surface markers**. Cells were stained with specific monoclonal antibodies. (A) Lymphocyte gate was drawn using forward and side scatter. (B) Lymphocytes were then plotted on CD19 APC against forward scatter. (C) CD19+ lymphocytes were plotted on CD19 APC against CD10 FITC, (D) CD19+ lymphocytes CD21 PE-cy5 against CD27 PE. Two additional gates were drawn on CD19^+^CD10^-^ and CD21^hi^CD27^-^ populations and plotted on CD21 PE-cy5 against CD27 PE (F**)** and CD21 PE-cy5 against CD10 FITC (E) to identify mature and immature B cells respectively. Using quadrant statistics, distinct populations of naïve (CD21^hi^CD27^-^), classical memory (CD21^hi^CD27^+^), activated memory (CD21^lo^CD27^+^), atypical memory (CD21^lo^CD27^-^), and immature transitional (CD10^+^CD21^hi^) B cells were identified.


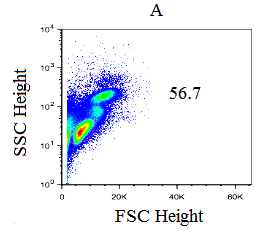

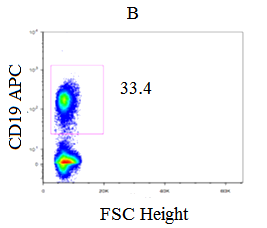

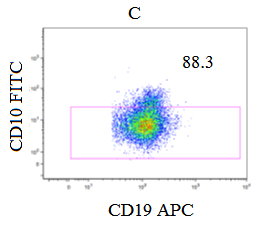


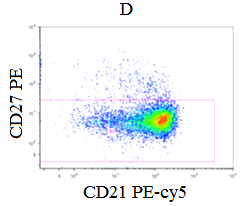

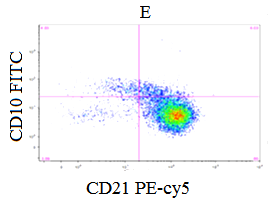

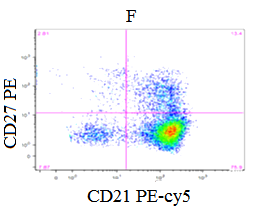


**S3 Fig**: **Proportion of B cells increased during the first two years of lif**e: Proportion of medians of CD4+ (CD3+CD4+) T cells, CD8+ (CD3+CD8+) T cells, B (CD19+) cells, NK (CD3-CD56+) cells and γδ (CD3+γδ+) T cells in healthy Malawians different of different age groups.


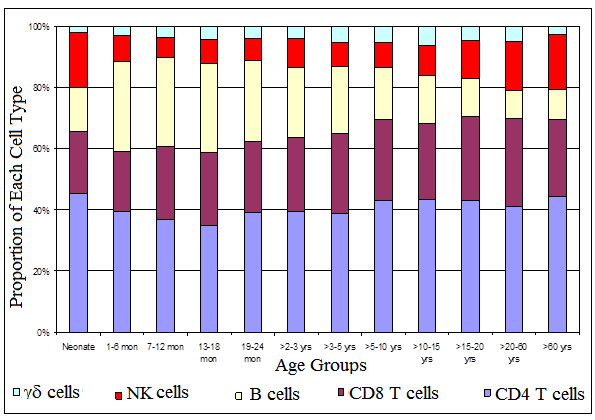


**SUPPLEMENTARY TABLES**

**S1 Table: List of Monoclonal Antibodies:** Catalogue Number and name of the supplier for the different monoclonal antibodies used in both portions of the study

| **Antibody** | **Product Number** | **Supplier** |
| --- | --- | --- |
| Anti-CD19-APC | 641395 | BD |
| Anti-CD27-PE | 340425 | BD |
| Anti-moGI-FITC | 555909 | BD |
| Anti-moGI-PE | 555749 | BD |
| Anti-moGI-PerCP | 555751 | BD |
| Anti-moGI- APC | 550795 | BD |
| Anti -CD19 APC | 555415 | BD Pharmingen, San Jose, California |
| Anti-CD21 PE-cy5 | 551064 | BD Pharmingen, San Jose, California |
| Anti-CD10 FITC | 11-0106-42 | eBiosciences, San Diego, California |
| Anti CD27 PE | 12-0271-82 | eBiosciences, San Diego, California |

1. BD = Becton Dickinson.
2. FITC = Fluorescein Isothiocyanate
3. PE = Phycoerythrin
4. PE-cy5 = Phycoerythrin-Cyanin5
5. PerCP = Peridinin chlorophyll protein
6. APC = Allophycocyanin

**S2 Table**: Medians (10^th^ and 90^th^ percentiles) of B cell subsets presented by age and gender

| **Group** | **Age Range** | **Sex** | **% B cells** | **% Naïve B cells** | **% Memory B cells** | **B cell Counts/μL** |
| --- | --- | --- | --- | --- | --- | --- |
| 1 | Neonates | F  (n = 37) | 11.83  (1.21 – 26.06) | 97.34  (70.72 – 99.33) | 2.66  (0.76 – 29.28) | 475  (27 – 1,646) |
|  |  | M  (n = 24) | 9.89  (2.68 – 25.15) | 96.24  (69.43 – 98.76) | 3.74  (1.24 – 30.57) | 326  (81 – 1,056) |
| 2 | 1 to 6 months | F  (n = 44) | 29.57  (11.4 – 48.69) | 95.94  (90.70 – 98.38) | 4.06  (1.62 – 9.21) | 1,631  (516 – 4,691) |
|  |  | M  (n = 24) | 26.11  (10.65 – 39.28) | 95.43  (54.25 – 98.03) | 4.35  (1.50 – 18.91) | 1,266  (291 – 3,415) |
| 3 | 7 to 12 months | F  (n = 31) | 28.42  11.66 – 46.71) | 89.94  (46.52 – 96.13) | 10.06  (3.87 – 24.46) | 2,074  (641 – 4,975) |
|  |  | M  (n = 35) | 28.49  (7.25 – 51.59) | 91.77  (80.85 – 96.12) | 8.23  (3.88 – 19.15) | 1,902  (284 – 5,933) |
| 4 | 13 to 18 months | F  (n = 15) | 28.05  (18.16 – 71.58) | 85.76  (21.43 – 95.84) | 13.19  (0.26 – 23.790 | 1,732  (808 – 4,800) |
|  |  | M  (n = 27) | 26.95  (17.06 – 48.62) | 89.13  (77.20 – 94.49) | 10.87  95.51 – 22.80) | 1,590  (732 – 3,609) |
| 5 | 19 to 24 months | F  (n = 16) | 27.46  (11.97 – 42.84) | 83.01  (60.12 – 97.57) | 17.14  (2.46 – 39.88) | 1,100  (646 – 2,3820 |
|  |  | M  (n = 33) | 24.89  (15.80 – 44.74) | 84.61  (73.71 – 93.08) | 15.44  (6.92 – 30.12) | 1,362  (566 – 4,973) |
| 6 | >2 to 3 years | F  (n = 20) | 23.53  (5.90 – 33.27) | 78.10  (64.19 – 96.66) | 21.90  (3.34 – 35.81) | 1,053  (130 – 1,545) |
|  |  | M  (n = 28) | 22.34  (8.10 – 42.25) | 77.00  (34.02 – 94.98) | 21.72  (3.13 – 31.98) | 1,008  (227 – 3,211) |
| 7 | >3 to 5 years | F  (n = 22) | 19.89  (13.54 – 32.42) | 76.22  (60.62 – 90.87) | 23.78  (9.13 – 39.15) | 692  (406 – 1,486) |
|  |  | M  (n = 29) | 21.90  (13.20 – 34.79) | 76.61  (59.74 – 88.15) | 23.39  (12.08 – 40.12) | 686  (330 – 2,574) |
| 8 | >5 to 10 years | F  (n = 30) | 16.56  (5.77 – 34.60) | 73.44  (54.72 – 92.05) | 24.99  (7.95 – 44.80) | 568  (224 – 2,145) |
|  |  | M  (n = 26) | 15.97  (10.14 – 29.54) | 70.87  (58.47 – 88.69) | 29.13  (11.31 – 41.53) | 532  (229 – 1,081) |
| 9 | >10 to 15 years | F  (n = 36) | 13.98  (10.46 – 25.90) | 73.01  (51.59 – 85.26) | 26.90  (14.74 – 48.57) | 367  (200 – 932) |
|  |  | M  (n = 17) | 17.30  (10.82 – 24.37) | 73.00  (55.54 – 91.23) | 27.00  (8.77 – 44.17) | 468  (218 – 878) |
| 10 | >15 to 20 years | F  (n = 29) | 11.93  (6.83 – 19.21) | 66.60  (48.39 – 83.15) | 33.40  (16.85 – 51.61) | 251  (117 – 699) |
|  |  | M  (n = 29) | 11.79  (6.78 – 19.70) | 74.81  (48.14 – 90.60) | 29.19  (9.40 – 51.86) | 255  (71 – 531) |
| 11 | >20 to 60 years | F  (n = 33) | 9.32  (5.76 – 21.80) | 63.89  (43.59 – 8261) | 36.11  (17.39 – 56.41) | 218  (102 – 676) |
|  |  | M  (n = 20) | 7.47  (4.36 – 14.05) | 60.05  (28.28 – 72.35) | 39.95  (27.65 – 71.72) | 150  (100 – 261) |
| 12 | >60 years | F  (n = 31) | 10.56  (5.12 – 17.73) | 63.09  (34.43 – 88.20) | 36.91  (11.80 – 65.57) | 232  (122 – 608) |
|  |  | M  (n = 24) | 7.85  (2.96 – 20.82) | 54.95  (16.61 – 82.80) | 45.05  (17.20 – 83.39) | 184  (71 – 396) |
